# n-3 PUFAs for depression: treatment effect or absence-of-placebo effect?

**DOI:** 10.1038/s41398-022-02072-z

**Published:** 2022-07-26

**Authors:** Arian Memarpouri, Willem van der Does, Marc L. Molendijk

**Affiliations:** 1 Clinical Psychology Department, Leiden University, Leiden, The Netherlands; 2 Cognitive Psychology Department, Leiden University, Leiden, The Netherlands; 3 Leiden University Treatment and Expertise Center (LUBEC), Leiden, The Netherlands; 4 Leiden Institute of Brain and Cognition, Leiden University Medical Center, Leiden, The Netherlands

**Keywords:** Scientific community, Depression

Dear Editor,

In a meta-analysis, Liao et al. [1]. conclude that n-3 PUFAs have a therapeutic effect on depression. Here we argue that this conclusion is premature.

Firstly, in contrast with their stated inclusion criteria, the data from 2 trials [2, 3] did not include an inert placebo arm. Also in contrast with an inclusion criterion, another trial was carried out in a non-(clinically) depressed sample [4]. Furthermore, data from a single trial were included twice [5, 6] and placebo conditions of this trial, and that of one other trial [7], were double-counted in analyses. The use of dependent data in meta-analyses gives a false impression of precision [8].

We carried out a random-effect meta-analysis on the corrected dataset, which yielded a Standardized Mean Difference (SMD; Cohen’s *d*) of −0.31 (95% Confidence Interval (CI) = −0.57 to −0.05) favoring n-3 PUFAs over placebo in the treatment of depression. Liao et al. report an SMD of −0.26 (95% CI = −0.47 to −0.09). Consequently, the errors do not invalidate the study conclusion, but note that the CI has widened.

Liao et al. [1]. included a trial performed by Marangell et al. [9], who in their report had presented a null-finding on the differences in the efficacy of n-3 PUFAs versus placebo in the treatment of depression (*P* = 0.43, page 996). In the meta-analysis, the same RCT is presented as having a large and statistically significant effect (SMD = −0.82). This mismatch is due to the parameter for efficacy that Liao et al. [1]. used: *data at a single point at the end of the trial*. This parameter is inferior to the crucial time x treatment interaction. Marangell et al. [9]. indeed report a large difference between the treatment and placebo group post-treatment; however, this is due to the fact that randomization failed. The control group scored significantly higher on the primary outcome measure at baseline, and this difference was not affected by treatment. To present this particular RCT as a positive trial is contrary to the findings reported in the original paper.

We noticed that the apparent positive effect of n-3 PUFAs was partly driven by a low or absent placebo effect. In antidepressant trials, placebo responses are typically higher on interviewer-rated scales (SMD = −1.85; 95% CI = −2.01 to −1.69) relative to self-report (SMD = −0.67; 95% CI = −0.85 to −0.49) [10]. We calculated the pooled treatment effect (interview-based) in the (active) n-3 PUFAs treatment arms and found it to be −1.36 (95% CI = −1.75 to −0.97). Consequently, the effect of n-3 PUFAs on depression outcome appears lower than the effect of placebos in antidepressant treatment trials. In this particular meta-analysis [1], the smallest placebo responses were observed in the four included studies performed in Iran [7, 11–13]. In fact, placebo responses reported in trials from Iran (SMD = −0.18; 95% CI = −0.44 to 0.06) are lower (*P* < 0.001) than those observed in non-Iran-based trials (SMD = −1.34; 95% CI = −1.73 to −0.96). There is no difference in the weighted average response to n-3 PUFAs treatment in Iranian versus non-Iranian studies (SMD’s of −1.32 and −1.34 respectively, *P* = 0.94). Consequently, the Iranian findings are driven by a lack of response in the placebo condition. The size of the placebo effect may depend on many factors, and a direct comparison between n-3 PUFAs studies and antidepressant studies is hazardous. Still, placebo effects have been demonstrated even with ‘open-label placebo’ [14, 15], rendering its absence in Iranian studies hard to explain. The authors of these RCTs did not respond to our requests for information. It may be interesting to note that only eight of 24 effect sizes in the meta-analysis show that n-3 PUFAs outperform placebo, without considering substantial publication bias. Six of these significant studies yielded unexpected low placebo responses (i.e., SMD’s < 0.4) [the Iranian studies and refs. [16, 17]].

Notably, the results from Iranian studies put a stamp on the outcomes of the meta-analysis. When excluded from the analysis, the overall result of the meta-analysis (following the approach by Liao et al. [1]) is a null-finding (SMD = −0.11; 95% CI = −0.36 to 0.10). Also, in case proper effect-size estimates are imputed for the Iranian studies (e.g., SMD = −1.00), a random-effects model over corrected data yields a null effect (SMD = −0.18; 95% CI = −0.40 to 0.04).

We argue that the quality of the underlying data reported by Liao et al. [1]. does not allow the conclusion that n-3 PUFAs demonstrate a therapeutic effect on depression. This is in line with a recent Cochrane review showing a lack of treatment effects of n-3 PUFAs on depression [18].
